# Iron deficiency disrupts embryonic haematopoiesis but not the endothelial to haematopoietic transition

**DOI:** 10.1038/s41598-019-42765-y

**Published:** 2019-04-23

**Authors:** Maya Shvartsman, Saygın Bilican, Christophe Lancrin

**Affiliations:** European Molecular Biology Laboratory, EMBL Rome, Epigenetics and Neurobiology Unit, Via Ramarini 32, 00015 Monterotondo, Italy

**Keywords:** Stem cells, Haematopoiesis

## Abstract

In this study, we aimed to explore how cellular iron status affects embryonic haematopoiesis. For this purpose, we used a model of mouse embryonic stem cell differentiation into embryonic haematopoietic progenitors. We modulated the iron status by adding either the iron chelator Deferoxamine (DFO) for iron deficiency, or ferric ammonium citrate for iron excess, and followed the emergence of developing haematopoietic progenitors. Interestingly, we found that iron deficiency did not block the endothelial to haematopoietic transition, the first step of haematopoiesis. However, it did reduce the proliferation, survival and clonogenic capacity of haematopoietic progenitors. Surprisingly, iron deficiency affected erythro-myeloid progenitors significantly more than the primitive erythroid ones. Erythro-myeloid progenitors expressed less transferrin-receptor on the cell surface and had less labile iron compared to primitive erythroid progenitors, which could reduce their capacity to compete for scarce iron and survive iron deficiency. In conclusion, we show that iron deficiency could disturb haematopoiesis at an early embryonic stage by compromising more severely the survival, proliferation and differentiation of definitive haematopoietic progenitors compared to restricted erythroid progenitors.

## Introduction

Embryonic haematopoiesis is an essential and complex process, which supplies blood to the developing embryo and the adult. It generates a wide range of haematopoietic progenitors and stem cells (HPSCs), from the primitive non-self-renewing erythroid progenitors of the yolk sac to the long-term self-renewing haematopoietic stem cells, which will reside in the adult bone marrow and continuously generate all blood lineages^1,2^. Despite its overall complexity, embryonic haematopoiesis can be simplified into a sequence of steps common for all types of HPSCs. The first step, the endothelial-to-hematopoietic transition (EHT), initiates blood development whereby endothelial cells of a particular type called haemogenic endothelium undergo significant morphological and transcriptomic changes to become HPSCs^3–5^. Afterwards, the HPSCs proliferate, differentiate, and migrate to colonize the foetal liver and bone marrow^2,6–8^. Recent work on human^9^ and mouse embryonic stem cells^10,11^ as well as on reprogrammed mouse embryonic fibroblasts^12^ contributed significantly to our knowledge of how transcription factors and growth factors control embryonic haematopoiesis. Yet the role of iron in the process of embryonic haematopoiesis is not completely understood.

Iron is an essential micronutrient required for catalysis, DNA synthesis, redox reactions and oxygen transport^13^. Iron deficiency through a knockout of iron import proteins like transferrin receptor (Tfrc) or Dmt1 (Slc11a2) causes anaemia and embryonic or early postnatal lethality in mice^14,15^. Nutritional iron deficiency in pregnant females increases the risk of iron deficiency and iron deficiency anaemia in the offspring, according to animal models and human epidemiological studies^16,17^. Hypotransferrinaemic hpx/hpx mice^18^ or mice chimeric for Tfrc knockout^19^ have a defect in T lymphoid differentiation, suggesting that the effects of iron deficiency might not be restricted only to the erythroid lineage. We therefore hypothesized that iron was important for an early step in embryonic haematopoiesis, which is common for all developing blood cells. Thus, we investigated the impact of iron on the process of EHT and early HPC populations.

To dissect the step of embryonic haematopoiesis when iron is most required, we needed an experimental model of embryonic haematopoiesis where we could change the cellular iron status quickly and reversibly and measure the effect in real time. To fulfil these requirements, we chose the experimental model of mouse embryonic stem cells progressively differentiating into blood cells through a haemangioblast stage^3^, similarly to what happens in yolk sac haematopoiesis^3^. The haemangioblast stage cultures start as Flk1^+^ mesoderm and differentiate into a mixed culture of endothelial, haematopoietic progenitor, and vascular smooth muscle cells^3^. In these cultures, we modified the cellular iron status by adding either an iron chelator (DFO) to cause iron deficiency or adding ferric ammonium citrate to induce a state of iron excess^20^.

In this work, we demonstrated that iron deficiency by DFO did not block EHT itself, but it differentially affected the proliferation, survival and differentiation of early haematopoietic progenitors. In contrast, iron excess had no adverse effects on haematopoietic progenitors. Thus, our findings offer broader understanding of how iron deficiency could affect embryonic haematopoiesis^16^.

## Results

### Iron deficiency by DFO does not inhibit EHT

We first tested whether iron deficiency would block the first step of embryonic haematopoiesis, which is the endothelial-to-haematopoietic transition (EHT)^3–5^. This hypothesis was based on previously published evidence that iron chelators were inhibiting the epithelial-to-mesenchymal transition (EMT), a mechanism similar to EHT^21^.

**Figure 1:**
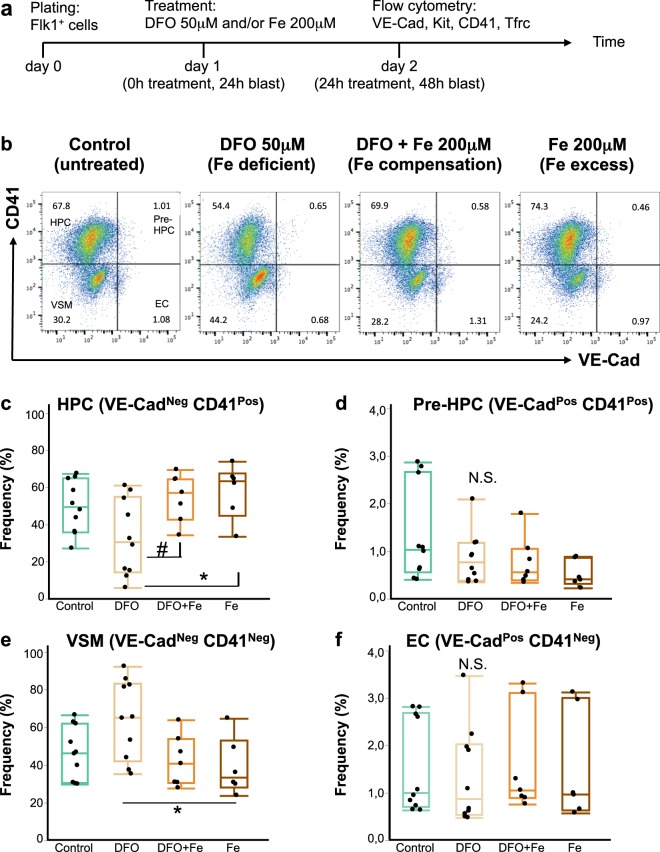
Iron deficiency does not block endothelial to haematopoietic transition. (**a**) A schematic timeline of an experiment in blast culture. Flk1^+^ cells were plated in blast mix on day 0, treated 24 hours later and profiled by flow cytometry for expression of endothelial and hematopoietic markers after another 24 hours. The treatments were: nothing (control), DFO 50 μM (iron-deficient conditions), DFO + ferric ammonium citrate 200 μM (simultaneous neutralization of iron deficiency, or iron compensation), Fe 200 μM (ferric ammonium citrate for iron excess conditions). (**b**) A flow cytometry plot showing expression of CD41 against VE-Cad in day 2 blast (24 hours of treatment). According to the expression of these markers, each blast culture can be divided into 4 quadrants: VE-Cad^−^ CD41^−^ = vascular smooth muscle cells (VSM); VE-Cad^+^ CD41^−^ = endothelial cells (EC); VE-Cad^+^ CD41^+^  = pre-hematopoietic progenitor cells (Pre-HPC); VE-Cad^−^ CD41^+^  = hematopoietic progenitor cells (HPC). The experimental conditions are shown from left to right. The Tukey’s boxplots of the different cell type frequencies from flow cytometry experiments are shown for HPC (**c**), Pre-HPC (**d**), VSM (**e**) and EC (**f**). Box whiskers show minimum and maximum, the line inside boxes shows median. For control and DFO groups n = 10; for DFO + Fe and Fe groups n = 6. All groups were analysed by one-way ANOVA followed by Tukey’s multiple comparisons test. For (**c**) ^#^p = 0.049 and *p = 0.027, for **(e)** *p = 0.032, for **(d,f**) N.S. = not significant.

For this purpose, we differentiated mouse embryonic stem cells^22^ into hemangioblast cultures^3,10,11^ and followed the formation of haematopoietic progenitors from hemangioblast as a function of iron status and time. The time course of our experiments is schematically represented in Fig. 1a. Our hemangioblast cultures were composed of four major cell types as demonstrated in Fig. 1b: vascular smooth muscle cells (VSM), endothelial cells (EC), haematopoietic progenitor cells (HPCs) and cells in an intermediate EHT stage, referred to as Pre-HPCs. These four cell types were distinguished in flow cytometry by expression of an endothelial marker VE-Cadherin (VE-Cad) and an early haematopoietic marker CD41. Cells expressing neither VE-Cad nor CD41 were vascular smooth muscle cells; endothelial cells, VE-Cad^+^ CD41^−^; HPCs, VE-Cad^−^ CD41^+^ and Pre-HPCs, VE-Cad^+^ CD41^+^.

If iron plays an essential role in EHT, we would expect that the addition of an iron chelator to our hemangioblast cultures would reduce HPCs frequency and cause accumulation of endothelial cells and/or Pre-HPCs^7^. DFO addition at 50 μM for 24 hours reduced the frequency and the absolute cell number of HPCs in culture (Fig. 1b,c, Supplementary Fig. 1). However, neither the frequency nor the cell number of endothelial and Pre-HPCs were increased by DFO (Fig. 1b,d,f, Supplementary Fig. 1), suggesting no accumulation of these cells took place. We further observed a slight increase in the frequency of vascular smooth muscle cells after DFO treatment (Fig. 1e), but their absolute cell number was not increased (Supplementary Fig. 1).

Excess iron added as 200 μM ferric ammonium citrate together with DFO abrogated all effects of DFO in culture, demonstrating that the observed effects were truly due to iron deficiency. Iron addition together with DFO or alone did not significantly reduce the frequencies and absolute cell numbers of any cell type compared to untreated control (Fig. 1, Supplementary Fig. 1), suggesting that in our conditions the given iron concentration was not toxic to cells. The 200 μM of iron should already be in excess to saturate the total transferrin in our culture medium, since we estimate the total concentration of transferrin in our culture medium as ~10 μM. Our estimation is based on average 140 μM total iron binding capacity of bovine serum^23^ (equivalent to ~70 μM transferrin^24^) plus contribution from human holotransferrin and conditioned medium in our culture.

### Iron deficiency differentially affects haematopoietic progenitors

**Figure 2:**
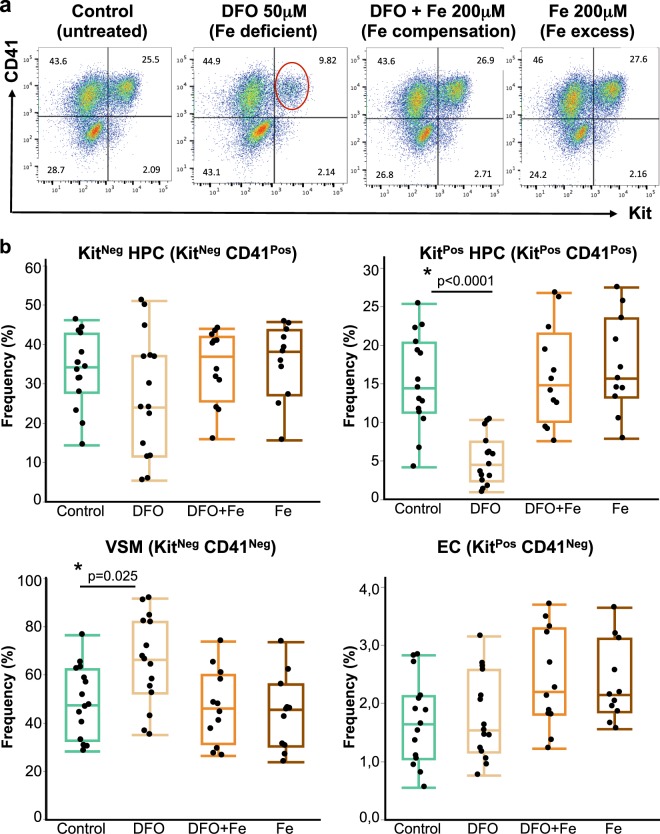
Iron deficiency reduces the frequency of Kit^Positive^ Haematopoietic Progenitor Cells. (**a**) A flow cytometry plot showing expression of CD41 against Kit in day 2 blast (24 hours of treatment). According to the expression of these markers, each blast culture can be divided into 4 quadrants: Kit^−^ CD41^−^ = VSM; Kit^+^ CD41^−^ = EC; Kit^+^ CD41^+^  = Kit positive HPC (Kit^Pos^ HPC); Kit^−^ CD41^+^  = cKit negative HPC (Kit^Neg^ HPC). The experimental conditions are shown from left to right. The red circle shows the Kit^Pos^ HPC population whose frequency decreased following the DFO treatment. (**b**) Tukey’s boxplots of cell type frequencies from flow cytometry experiments. Box whiskers show minimum and maximum, the line inside boxes shows median. For control and DFO groups n = 15; for DFO + Fe group n = 13 and Fe group n = 11. All groups were analysed by one-way ANOVA followed by Tukey’s test with p values shown on graphs.

All haematopoietic progenitors in our cultures can be divided into two major subtypes: definitive Kit^Pos^ HPCs (Kit^+^ CD41^+^) and primitive Kit^Neg^ HPCs (Kit^-^ CD41^+^). The Kit^Pos^ HPCs mostly yield multilineage haematopoietic colonies when plated on methylcellulose with appropriate growth factors and are capable of reconstituting irradiated mice albeit transiently, while the Kit^Neg^ HPCs yield mostly primitive erythroid colonies on methylcellulose and have no reconstitution capacity^3,25^. When we examined the effect of DFO on both kinds of HPCs, we saw that the frequency of Kit^Pos^ HPCs was significantly (p < 0.0001) reduced compared to the untreated control (Fig. 2a,b) and the frequency of Kit^Neg^ HPCs was not significantly changed. The cell numbers of both progenitor types were significantly reduced, but the decrease in Kit^Pos^ HPCs was stronger (Supplementary Fig. 2). It was surprising to observe a differential effect of iron deficiency on hematopoietic progenitors at such an early stage of development. Endothelial and vascular smooth muscle cells, viewed in these experiments as Kit^+^ CD41^−^ and Kit^−^ CD41^−^ respectively, behaved similarly to the experiments described in Fig. 1 (Fig. 2 and Supplementary Fig. 2). Since iron excess could also be potentially toxic to developing blood cells^26^, we also studied the effect of iron excess on hematopoietic progenitor formation. Excess concentrations of iron in a range of 0.2–5 mM ferric ammonium citrate caused no significant reduction of frequency or cell number in any cell type compared to untreated control. However, some reduction of HPCs cell number occurs at 10 mM iron suggesting that this concentration already starts being toxic to these cells (Supplementary Tables 1 and 2). The frequency and cell number of vascular smooth muscle and endothelial cells were not significantly affected at any iron concentration (Supplementary Tables 1 and 2). However, after performing an apoptosis analysis^27^, we found that 10 mM iron concentration increased the frequency of both apoptotic AnnexinV^+^ 7AAD^+^ and pre-apoptotic AnnexinV^+^ 7AAD^−^ cells after 24 hours treatment. Specifically, the frequency of apoptotic vascular smooth muscle cells and Kit^neg^ HPCs was significantly increased compared to untreated control or Fe 1 mM. The lower iron concentration of 1 mM gave no significant increase of apoptotic and pre-apoptotic cells after 24 hours of treatment (Supplementary Tables 3 and 4), suggesting that at this time point iron is only toxic to cells at concentrations as high as 10 mM.

### Iron deficiency does not affect haematopoietic progenitor identity

**Figure 3:**
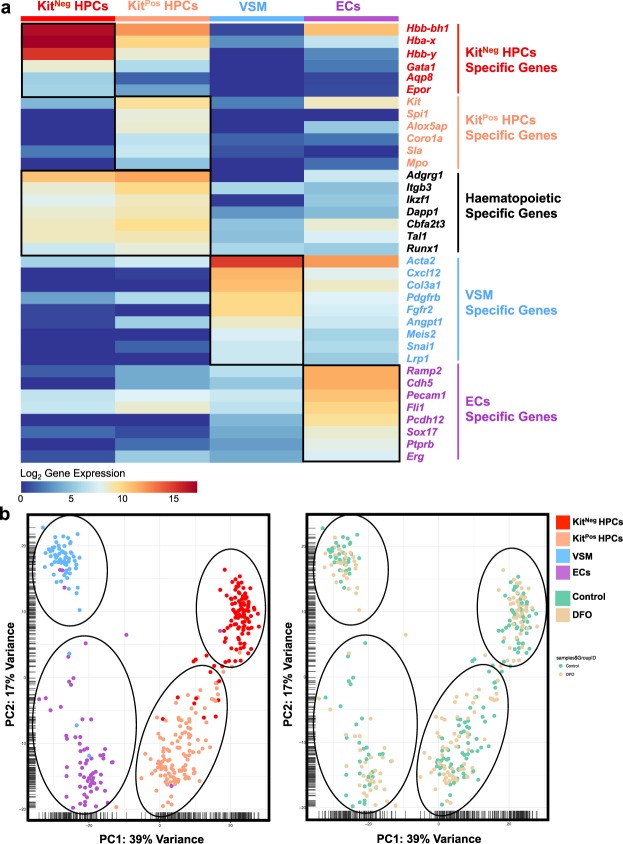
Iron deficiency does not change the identity of haematopoietic progenitors. (**a**) Heatmap showing the expression of key genes for the indicated populations found in control condition. The four groups were based on the phenotype of sorted single cells. (**b**) PCA plots showing the 366 cells tested by sc-q-RT-PCR (Control and DFO). The PCA plot on the left shows the four major cell clusters and the PCA on the right shows the distribution of cells from Control and DFO experimental conditions.

To find an explanation for the observed differential effect of iron deficiency on HPCs, we profiled gene expression in both kinds of progenitors by single-cell quantitative RT-PCR (sc-q-RT-PCR). We isolated single cells from Kit^Neg^ HPCs, Kit^Pos^ HPCs, VSM and ECs and tested a panel of 96 genes, among them endothelial (*Cdh5*, *Ramp2*, *Sox17* and *Erg*), haematopoietic (erythroid and myeloid), smooth muscle (*Acta2*, *Col3a1*, *Pdgfrb*, *Meis2* and *Snai1*) and iron metabolism (*Fth1*, *Tfrc* and *Slc11a2*) genes (Supplementary Fig. 3). Interestingly, the four populations defined by Kit and CD41 cell surface markers segregated into four distinct cell clusters following hierarchical clustering and Principal Component Analysis (PCA) analysis (Fig. 3 and Supplementary Fig. 3). The Kit^Neg^ HPCs were clearly erythroid, expressing genes of embryonic hemoglobins (*Hbb-y*, *Hbb-bh1* and *Hba-x*), *Epor*, *Gata1* and *Aqp8*. In contrast, the Kit^Pos^ HPCs were composed of cells expressing erythroid genes (albeit at a lower level than Kit^Neg^ HPCs) and others expressing white blood specific genes (*Spi1*, *Alox5ap*, *Coro1a* and *Mpo*). All HPCs and non-haematopoietic cells highly expressed the *Fth1* gene, which encodes one of the ferritin protein subunits.

Remarkably, DFO-treated cells of any cell type clustered together with its respective control cells, suggesting that iron deficiency had no large-scale effect on the expression profile of the selected gene panel as shown by hierarchical clustering, PCA and ANOVA pairwise analysis (Fig. 3, Supplementary Figs 3 and 4).

### Iron deficiency differentially affects proliferation and survival of haematopoietic progenitors

**Figure 4:**
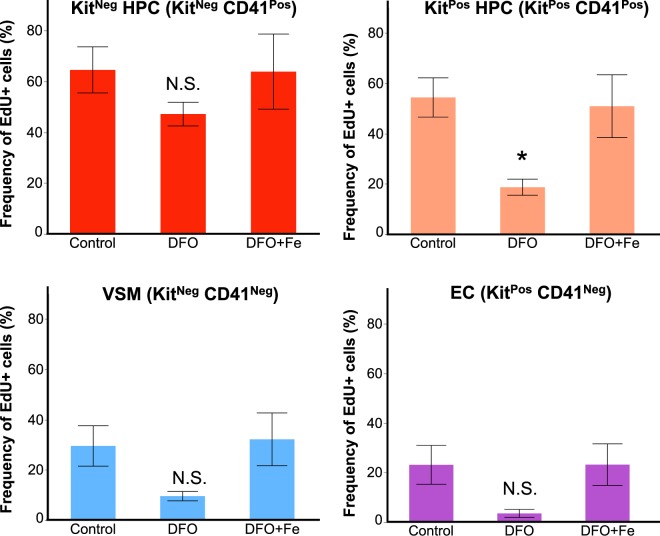
Iron deficiency reduces the proliferation of Kit^Pos^ HPCs. The frequency of EdU^+^ cells (i.e. in S-phase) is shown as a function of treatment for each of the cell types in the blast culture. Data are shown as mean ± SEM, n = 4. *p < 0.05, one-way ANOVA and Tukey’s multiple comparisons test.

Since iron deficiency by DFO selectively reduced the frequency of Kit^Pos^ HPCs, we investigated the causes of this reduction. Since EHT is not inhibited by the DFO treatment (Fig. 1), we hypothesized that the Kit^Pos^ HPCs frequency could be reduced because of a decrease in proliferation or an increase in cell death. We measured the cell proliferation in control, iron deficient and iron-excess conditions with the aid of a ClickIt-EdU kit, which only labels cells in S phase through incorporation of EdU^11^. Overall, haematopoietic progenitors were the most proliferating cells in culture having between 54–64% of S-phase cells, while endothelial and vascular smooth muscle cells proliferated less, with 23–30% (Fig. 4 and Supplementary Table 5). In control conditions, both Kit^Pos^ and Kit^Neg^ HPCs had similar proliferation rate, but the decrease in proliferation after DFO was significantly stronger in Kit^Pos^ HPCs. Adding excess iron together with DFO did not alter cell proliferation levels compared to control group.

**Figure 5:**
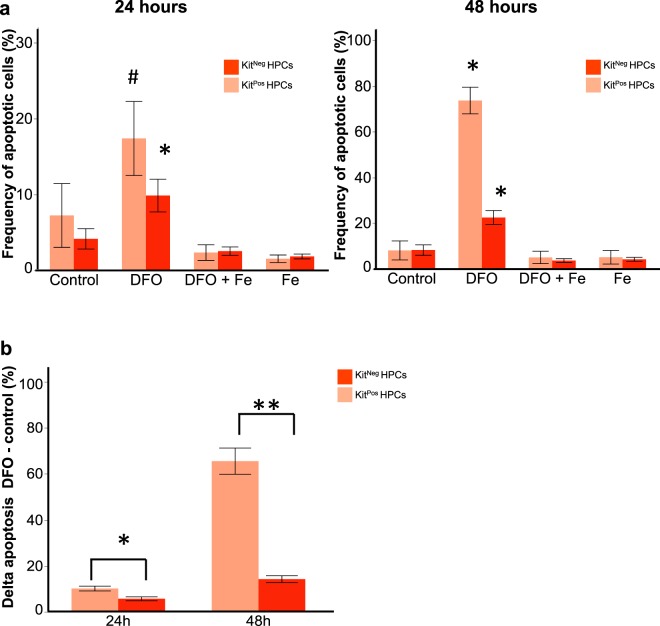
Iron deficiency leads to a higher apoptosis rate of Kit^Pos^ HPCs compared to Kit^Neg^ ones. (**a**) The frequency (%) of apoptotic (AnnexinV^+^ 7AAD^+^) cells is presented as a function of treatment in both Kit^Pos^ and Kit^Neg^ HPCs after 24 hours (left panel) and 48 hours (right panel) treatment. *p < 0.05 for control versus DFO or # for DFO versus Fe groups by one-way ANOVA and Tukey multiple comparisons test. (**b**) The delta apoptosis (% DFO - % control) was compared between Kit^+^ CD41^+^ and Kit^−^ CD41^+^ HPs. *p < 0.05 and **p < 0.01 for Kit^+^ CD41^+^ versus Kit^−^ CD41^+^ by paired two-tailed t-test.

Our apoptosis measurements using AnnexinV and 7AAD^27^ demonstrated that in control conditions the death rate of both progenitor types was not significantly different (Fig. 5a,b and Supplementary Table 6). DFO treatment increased the frequency of late apoptotic cells in both HPC types compared to control or iron-treated groups (Fig. 5a). This increase in AnnexinV^+^ 7AAD^+^ cells was seen at 24 and 48 hours of 50 μM DFO treatment. Excess iron added together with DFO displayed apoptotic cell frequencies close to control levels. The frequencies of pre-apoptotic AnnexinV + 7AAD- cells were not significantly changed by DFO (Supplementary Table 7). Since there was a baseline of cell death in control conditions, we calculated the net cell death as delta apoptosis by subtracting the frequency of apoptotic cells in control conditions from the frequency of apoptotic cells with DFO (Δ _DFO – control_). The net cell death was significantly higher in Kit^Pos^ HPCs than in Kit^Neg^ HPCs in all time-points of DFO treatment (Fig. 5b). Together, our results suggest that DFO reduces Kit^Pos^ HPCs frequency both by reducing proliferation and by increasing cell death.

### Iron deficiency reduces colony-forming unit capacity of both Kit^-^ and Kit^+^ haematopoietic progenitors

**Figure 6:**
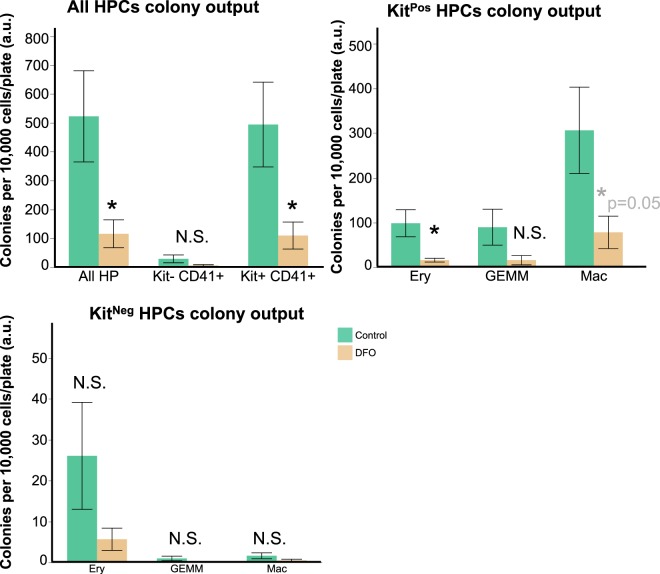
Iron deficiency reduces haematopoietic colonies’ formation. Colony formation in HPs isolated from control or DFO 50 μM-treated day 2 blast cultures. Data are presented as mean ± SEM, n = 5. Colonies were calculated per 10,000 cells/plate for all HPCs; Kit^Pos^ HPCs; Kit^Neg^ HPCs. *p < 0.05, paired two-tailed t-test; N.S. = non-significant control versus DFO.

We aimed to resolve whether iron deficiency would affect the differentiation of early haematopoietic progenitors into mature blood lineages. For this purpose, we sorted both kinds of HPs from control and DFO-treated conditions and seeded them on DFO-free methylcellulose plates for a week. As published previously^25^ and in concordance with the results of our sc-q-RT-PCR, Kit^Neg^ HPCs gave rise to mostly primitive erythroid colonies after one week on methylcellulose whereas the Kit^Pos^ HPCs gave rise to erythroid, erythromyeloid and macrophage colonies. The colony-forming unit (CFU) capacity of Kit^Pos^ HPCs was higher than of Kit^Neg^, in agreement with previously published work^3,25^. DFO treatment significantly reduced the total amount of colonies formed from all HPCs together (Fig. 6) and the amount of erythroid and macrophage colonies from Kit^Pos^ HPCs. We did not observe a statistically significant effect of DFO on colony output from Kit^Neg^ HPCs. Overall, DFO treatment of early HPCs for 24 hours was sufficient to compromise HP clonogenic capacity, disturbing differentiation in the longer term.

### Haematopoietic progenitors differ in their endogenous transferrin receptor protein expression and labile iron levels

**Figure 7:**
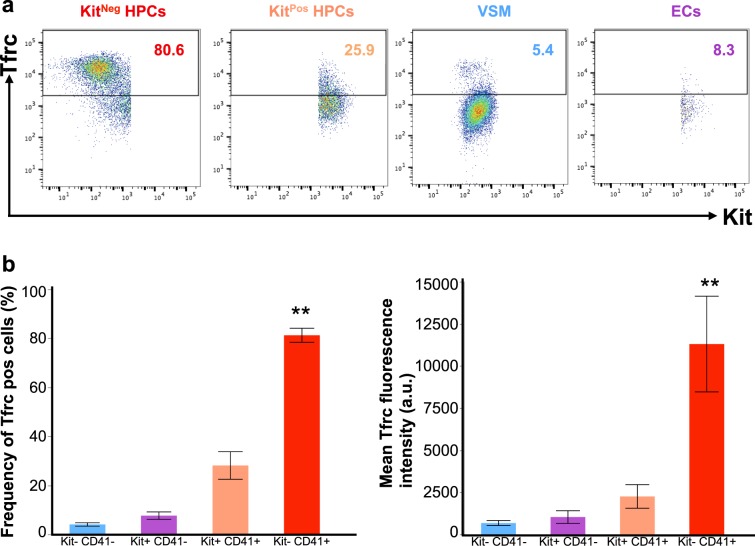
Kit^Neg^ HPCs express the highest level of Transferrin receptor. (**a**) Representative flow cytometry plots showing Tfrc (CD71) versus Kit expression in different cell types. (**b**) Frequency (%) of cells being positive for Tfrc in different cell types. Data are shown as mean ± SEM. **Significant in Kit^Neg^ HPCs versus Kit^Pos^ HPCs at p < 0.01, ANOVA and Tukey-Kramer. (**c**) Mean Tfrc fluorescence in arbitrary units in different cell types. **Significant in Kit^Neg^ HPCs versus Kit^Pos^ HPCs at p < 0.01, ANOVA and Tukey-Kramer.

We hypothesized that the haematopoietic progenitors the most sensitive to iron deficiency should have higher metabolic demand for iron, reflected by higher expression of transferrin receptor (Tfrc) protein on the cell surface. However, our flow cytometry studies paradoxically showed the opposite. The more sensitive Kit^Pos^ HPCs had significantly lower Tfrc expression than Kit^Neg^ HPCs, as seen by both the frequency (%) of Tfrc^+^ cells and the mean Tfrc fluorescence (Fig. 7 and Table 1). Overall in culture, haematopoietic progenitors had higher Tfrc expression than non-haematopoietic cells, and most of the Tfrc fluorescence came from Kit^Neg^ HPCs. Iron deficiency or iron excess gave a trend of increase or decrease of Tfrc expression respectively, but the Tfrc level was always higher in Kit^Neg^ HPCs compared to other cell types (Table 1). The Kit^Neg^ HPCs also had the highest labile iron levels in control culture as detected by cytosolic calcein (Table 1 and Supplementary Table 8). Together, these observations could explain our paradox of HPCs sensitivity to iron deficiency: the more sensitive Kit^Pos^ HPCs express less Tfrc and have less labile iron inside, therefore are less able to compete for scarce iron and survive through iron deficiency.

**Table 1:** Kit^Neg^ HPCs have highest Tfrc and labile iron compared to other cell types.

Cell type	Mean Tfrc fluorescence (a.u.)				Basal labile iron (r.u.)
	Control	DFO	DFO + Fe	Fe	Control
VSM	672 ± 152	786 ± 183	485 ± 80	482 ± 91	0.09 ± 0.02
EC	1026 ± 377	880 ± 130	429 ± 113	439 ± 126	0.22 ± 0.01
Kit^Pos^ HPCs	2253 ± 699	2529 ± 423	634 ± 124	765 ± 176	0.20 ± 0.02
Kit^Neg^ HPCs	11301 ± 2841*	12889 ± 2301*	2325 ± 438*^#^	2480 ± 552 *^#^	0.45 ± 0.1*

Transferrin receptor levels on the cell surface were measured by flow cytometry as described in Methods. Data are presented as mean ± SEM, N = 4, one-way ANOVA and Tukey’s multiple comparisons test.

Cytosolic Labile iron levels in control untreated cells were measured with calcein by flow cytometry as described^20^. Data are presented as mean ± SEM, N = 3, one-way ANOVA and Tukey’s multiple comparisons test.

*Significant in Kit^Neg^ HPCs vs other cell types, p < 0.01.

^#^Significant in treatment vs control, p < 0.01.

## Discussion

In the present work, we aimed to explore how cellular iron status affects embryonic haematopoiesis. While previous studies^14,15^ demonstrated that iron deficiency causes anaemia, some other works in Tfrc^−/−^ chimeric mice or the atransferrinemic mice showed that iron deficiency could also affect non-erythroid lineages^18,19^. Even in the total Tfrc knockout mouse, which had anaemia and embryonic lethality by E12.5^14^, there still was some residual yolk-sac erythropoiesis, suggesting that some haematopoietic progenitors and stem cells could be more sensitive to iron deficiency than others. We therefore hypothesized that iron deficiency could affect not only erythroid development, but rather affect a haematopoietic step common to all blood lineages.

We used an *in vitro* model system of mouse embryonic haematopoiesis, which allowed us to visualize the early steps of the process in real-time^3,10,11^. It has the advantages of accessibly and reversibly modifying the cellular iron by chemical or pharmacological means, and circumventing complications like embryonic lethality.

The addition of DFO to our hemangioblast cultures did not cause a massive accumulation of endothelial or Pre-HPCs, which is a characteristic of TGFβ treatment or overexpression of key transcription factors^10,11^. Neither the frequency nor the cell number of endothelial cells were increased (Figs 1,2 and Supplementary Figs 1, 2). Therefore, we conclude that DFO does not block EHT per se.

In our cultures, we observed two kinds of haematopoietic progenitors, which differ by their Kit expression Kit^Pos^ and Kit^Neg^ HPCs. DFO had an unexpected differential effect on HPCs, reducing the frequency and cell number of Kit^Pos^ more than of Kit^Neg^ (Fig. 2). These early progenitors differed in their endogenous transcriptomic profile (Fig. 3) already at this early stage. The Kit^Pos^ HPCs were erythro-myeloid, while the Kit^Neg^ were primitive erythroid by their gene expression profile. That difference in gene expression was also consistent with the HPC differentiation profile: Kit^Pos^ HPCs gave mixed lineage colonies after a week of CFU assay (Fig. 6), and Kit^Neg^ HPCs gave mostly primitive erythroid colonies. Since the Kit^Neg^ HPCs had high expression of haemoglobin genes (Fig. 3), high expression of Tfrc (Fig. 7) and low clonogenic capacity (Fig. 6), this cell population could consist of primitive erythroid hematopoietic progenitors and differentiating primitive erythroid lineage precursors. Our observations were consistent with previously published works^3,10,11,25^. DFO treatment did not change the overall transcriptomic profile of any HPCs (Fig. 3) and it did not change the type of colonies generated on methylcellulose (Fig. 6), suggesting that iron deficiency does not affect cell identity or the direction of differentiation. The overall HPC differentiation capacity, reflected by colony number, was reduced by DFO (Fig. 6) presumably due to inhibition of proliferation and increased cell death (Figs 4,5 and Supplementary Tables 3, 4). While the effects on proliferation and cell death were apparent right after completion of 24 or 48-hours treatment, the consequences on differentiation were long lasting. We could therefore suggest that iron deficiency could have both short and long-term effects on haematopoiesis.

The differential sensitivity to DFO we observed between the different populations of HPCs could be the result of a combination of different factors such as the iron-import capacity of a cell, the iron content inside the cell and the metabolic requirement for iron. Although, we initially assumed that the more sensitive progenitors would be more erythroid, requiring more iron for haemoglobin synthesis, in fact the opposite was true. We unexpectedly found that the erythro-myeloid Kit^Pos^ HPCs were the most sensitive to iron deficiency. When we measured the Tfrc and iron levels in both HPC types, we found that more sensitive Kit^Pos^ HPCs had endogenously lower Tfrc expression and less intracellular iron than the Kit^Neg^ HPCs (Fig. 7 and Table 1). Having less Tfrc on the cell surface and less intracellular labile iron, the Kit^Pos^ HPCs could be less capable of competing for scarce iron compared to Kit^Neg^ and be less capable of surviving a period of iron deficiency (Table 1 and Supplementary Table 8).

Overall, we demonstrate for the first time that iron deficiency could affect haematopoiesis at an unexpectedly early embryonic stage. It is known from previous works that iron deficiency in pregnant rats reduced the total iron content in whole embryos^16^ and foetal liver^28^, as well as haematocrit, haemoglobin and RBC count in the offspring^16^. Our study suggests that iron deficiency could have consequences beyond erythropoiesis, also affecting developing myeloid cells in the embryo. Our work indicates that other aspects of haematopoiesis besides erythropoiesis need also to be assessed in cases of iron deficiency.

## Methods

### Maintenance and differentiation of mouse embryonic stem cells (mESCs)

We used the A2lox Cre mESC cell line, which was a kind gift from Michael Kyba^22^. The detailed procedure of cell maintenance and differentiation is described in detail elsewhere^11^. Briefly, ESCs were plated and passed on feeder cells (mouse embryonic fibroblasts) for 5 days in DMEM-Knockout medium (Gibco, 10829-018) supplemented with 15% foetal bovine serum (Gibco, #10270-42Q4972K), 1% L-Glutamine (Gibco, 25-030-024), 1% Penicillin-Streptomycin (Gibco, 15140-122), 1% non-essential amino acids (Gibco, 11140-035), 0.24% of 50 mM β-mercaptoethanol (Gibco, 31350-010) and 0.0024% of 1 mg/ml LIF (EMBL-Heidelberg). After this, cells were passaged on gelatin for two days to dilute feeders out. One gelatin passage was done in DMEM-ES, and the other in IMDM-ES made with IMDM medium (Lonza, BE12-726F).

After the gelatin passages, we plated the cells in a medium for embryoid bodies (EBs) at 0.3 × 10^6^ cells/10 cm petri dish (Sterilin). The EB medium was made of IMDM (Lonza, BE12-726F), 15% foetal bovine serum (Gibco, #10270-42G9552K), 1% Penicillin-Streptomycin (Gibco, 15140-122), 1% L-Glutamine, 0.6% holotransferrin (Roche, 10652202001), 0.0039% MTG (Sigma, M6145) and 50 μg/ml ascorbic acid (Sigma, A4544). After 3–3.25 days of EB culture, Flk1^+^ cells were sorted from EBs using rat anti-mouse Flk1-APC antibody (eBioscience, #17-5821-81) with magnetic anti-APC MACS beads (Miltenyi Biotec, #130-090-855). Following the sort, the purified Flk1^+^ cells (>95% purity) were frozen and stored in liquid nitrogen for further experiments.

### Hemangioblast cultures and experiments

Flk1^+^ cells were plated on gelatin-coated 6-well plates at 1 × 10^6^ cells/plate in a blast-mix medium containing IMDM (Lonza BE12-726F), 10% foetal bovine EB serum (Gibco, #10270-42G9552K), 0.6% (0.18 mg/ml) human holotransferrin of approximately 30% iron saturation (Roche, 10652202001), 0.0039% MTG (Sigma, M6145), 25 μg/ml ascorbic acid (Sigma, A4544), 15% D4T supernatant (EMBL Rome), 0.05% of 10 μg/ml VEGF (Peprotech, 100-20) and 0.1% of 10 μg/ml IL6 (Peprotech, 216-16). Our blast culture medium has transferrin from the 10% foetal bovine serum (estimated as 7 μM final concentration based on the average 140 μM total iron binding capacity of bovine serum^23^ and the 2:1 Fe:Trf binding stoichiometry^24^), from the 0.18 mg/ml human transferrin (estimated as 2 μM based on 80 kDa molecular weight for transferrin) and from the 1.5% foetal bovine serum derived from D4T conditioned medium (estimated as 1 μM). Overall, we estimate to have ~10 μM total transferrin in the cell culture medium. At 24 hours after plating, cells were either left untreated or supplemented with 50 μM deferoxamine (DFO, Sigma, D9533), 200 μM ferric ammonium citrate (Sigma, F5879), either alone or in combination with 50 μM DFO. The stock solutions of DFO and ferric ammonium citrate were prepared in sterile distilled water (Gibco) and were added to the cells at a volume, which would not exceed 2% of the total well volume. In some cases, we used a well supplemented with 1–2% water as a control, which was similar to a well with no addition at all (Shvartsman and Lancrin unpublished observations). The time course of haematopoiesis in culture was followed by IncuCyte (Essen Bioscience) time-lapse microscopy from the moment of treatment until 24 hours later. After 24 hours of treatment (unless indicated otherwise), cells were harvested with TrypLE, collected into IMDM with 10% FBS and stained for flow cytometry with one of the following combinations of rat anti-mouse antibodies from eBioscience: CD144-efluor660 (# 50-1441-82) and CD41-PE (# 12-0411-82); CD117-APC (# 17-1171-81) and CD41-PE; any of the combinations above plus CD71-biotin (BD Pharmingen, #557416) and streptavidin-AlexaFluor488 (Invitrogen, S11223). The antibody dilutions were 1:200 for CD144-efluor660, CD117-APC and CD71-biotin, 1:400 for CD41-PE. Streptavidin-AF488 was diluted 1:500. Dead cells were excluded by 7-AminoactinomycinD (7AAD, Sigma #A9400) 1:100 staining. Fluorescence was measured with the aid of FACSCanto flow cytometer (BD).

### Cell proliferation assay

Cell proliferation was measured with the Click-It Plus EdU Alexa Fluor 488 flow cytometry assay kit from Invitrogen (C10633), according to manufacturer’s instructions. Briefly, our blast cultures received 10 μM EdU for 1 hour before cell harvesting and staining with above-mentioned antibodies. Stained cells were fixed and permeabilized and the ClickIt reaction was performed by manufacturer’s instructions. Fluorescence was measured on FACS-Canto (BD).

### Apoptosis assay

Apoptosis was measured by AnnexinV-FITC apoptosis detection kit (eBioscience #88 8005 72) according to manufacturer’s instructions. Briefly, cells were harvested, stained with the anti-mouse cKit-APC and CD41-PE antibody mix, washed, incubated with Annexin-V-AF488 in AnnexinV-binding buffer, washed and put on ice. 7-AminoactinomycinD was used as a nuclear stain to label dead cells^27^. Fluorescence was measured on FACS-Canto (BD).

### Haematopoietic Colony Forming Unit (CFU) assay

Hemangioblast cultures were grown as described above, but in 20-cm gelatinized dishes (Corning #10314601) instead of 6-well plates. 24 hours after plating, cells were treated or not with DFO 50 μM for 24 hours, then harvested and stained with a mix of anti-mouse CD144-efluor660, CD117-APC and CD41-PE antibodies as described above. Haematopoietic progenitors were gated as VE-Cad^−^ CD41^+^ and sorted into separate sterile falcons according to their Kit/CD41 fluorescence profile (Kit^+^ CD41^+^ or Kit^-^ CD41^+^) with the FACSAria cell sorter. Dead cells were excluded by 7-AAD staining. The cells were plated into CFU mix with 55% methylcellulose, as previously described^3^. Colony number was counted 1 week later. The experiment was independently repeated 5 times.

### Single-cell quantitative RT-PCR

Hemangioblast cultures were grown as above and treated with either nothing or DFO for 24 hours. Cells were harvested and stained with the same combination of anti-mouse antibodies used for colony assays. Single cells from each cell type (Kit^−^ CD41^−^, Kit^+^ CD41^−^, Kit^+^ CD41^+^ and Kit^−^ CD41^+^) were sorted with the FACSAria fluorescence cell sorter (BD Biosciences) into 96-well plates filled with 2 x lysis buffer from the CellsDirect One Step qRT-PCR Kit (Invitrogen, # 11753100) and snap-frozen on dry ice. RT/Specific target amplification reaction (RT-STA) was performed according to manufacturer’s instructions. After RT-STA, individual single-cell qPCR was run on the Fluidigm Biomark HD system. Analysis was performed using R as previously described^11^.

### Labile iron measurements

The method for labile iron measurements with calcein green was adapted from Shvartsman *et al*.^20^. Briefly, cells were stained with calcein-green AM 0.03 μM for 10 min at 37 °C, washed, stained with antibodies as described above and sampled by flow cytometry in 10 min intervals before (baseline) and after addition of a cell permeable chelator (L1 50 μM). The relative labile iron content is measured as the normalized delta fluorescence (ΔF) between baseline and chelator addition^20^.

### Data analysis and statistics

Flow cytometry raw data were analysed by exclusion of debris and doublets, exclusion of dead 7AAD + cells, and gating on different types of live cells according to their VE-Cad/CD41 or Kit/CD41 fluorescence. Single-stained controls were used for compensation. Flow cytometry data were analysed using the FlowJo 10.2 software (TreeStar Inc.).

Data from flow cytometry experiments were plotted either as frequency (% of cells of a given cell type from total number of cells), or as absolute cell number (the frequency x total cell number (x10^4^)/well calculated by haemocytometer/100). Data were calculated as mean+/− standard deviation or standard error. Each experiment was independently performed from n = 3 to n = 15. Statistical analysis was either one-way ANOVA followed by Tukey-Kramer multiple comparisons test to compare multiple experimental groups, or paired two-tailed t-test to compare only control versus DFO. Graphs and statistical analysis were done by JMP Pro 12.1.0.

## Supplementary information

Supplementary Information
