# Supplementary material for: Iron deficiency disrupts embryonic haematopoiesis but not the endothelial to haematopoietic transition
Source: Sci Rep. 2019 Apr 23;9:6414. doi: 10.1038/s41598-019-42765-y (PMC6478831; doi:10.1038/s41598-019-42765-y)
Supplement: Supplementary file 1 — Supplementary Information [file 41598_2019_42765_MOESM1_ESM.pdf]

Supplementary Information

Iron deficiency disrupts embryonic haematopoiesis but not the endothelial to haematopoietic transition.

Maya Shvartsman[1], Saygın Bilican[1] and Christophe Lancrin[1]

[1] European Molecular Biology Laboratory, EMBL Rome, Epigenetics and Neurobiology Unit, Via Ramarini 32, 00015 Monterotondo, Italy

Supplementary Figure 1: Number of cells for populations defined by CD41 and VE-Cad expression

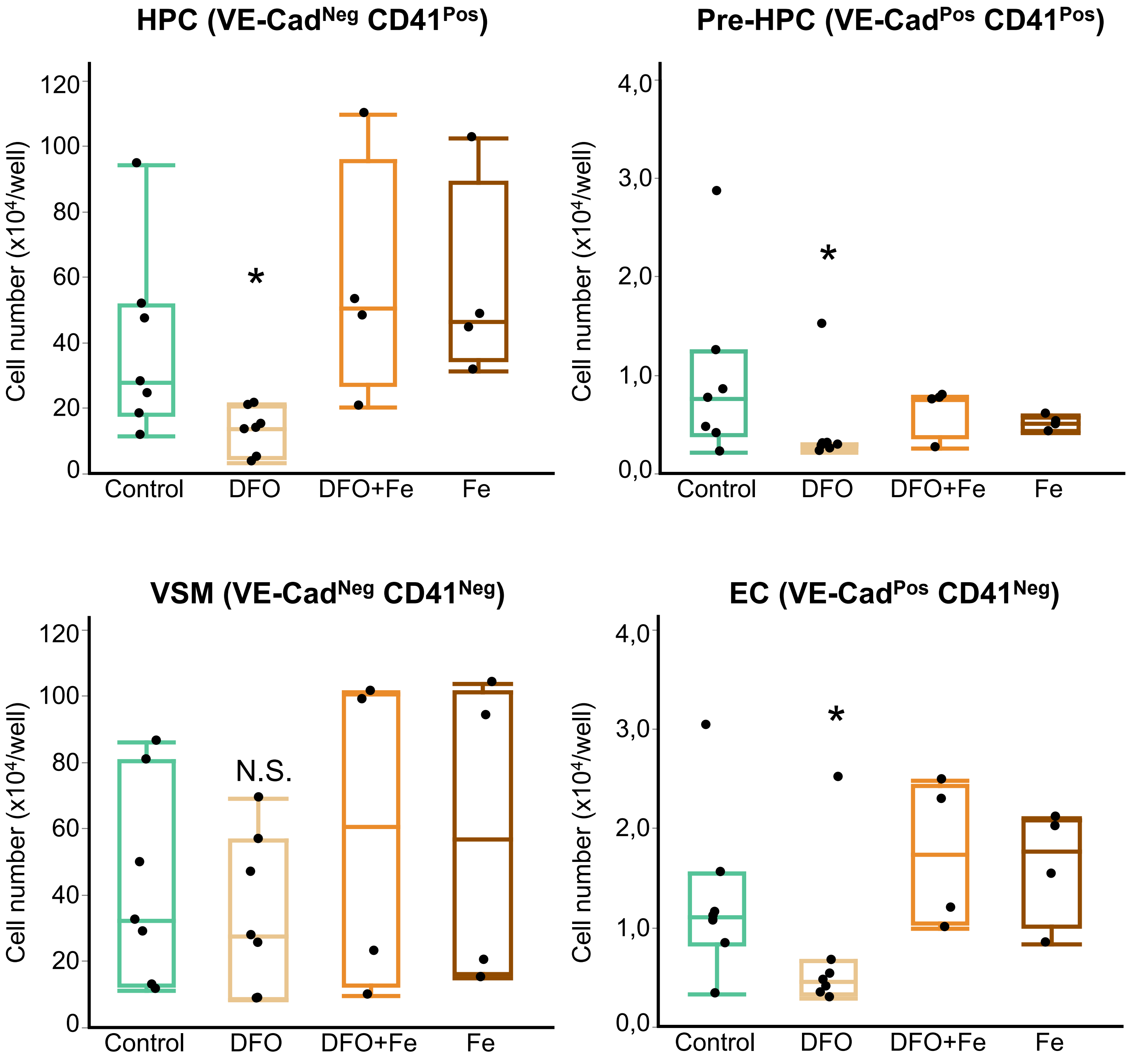

Tukey's boxplots of cell numbers calculated from flow cytometry experiments as cell type frequency * total cell number (10^4 cells/well)/100. Box whiskers show minimum and maximum, the line inside boxes shows median. For control and DFO groups N=7; for DFO+Fe and Fe groups N=4. Cell numbers in control versus DFO were analyzed by paired t-test. N.S. = not significant, * significant at $p<0.05$.

Supplementary Figure 2: Number of cells for populations defined by CD41 and Kit expression

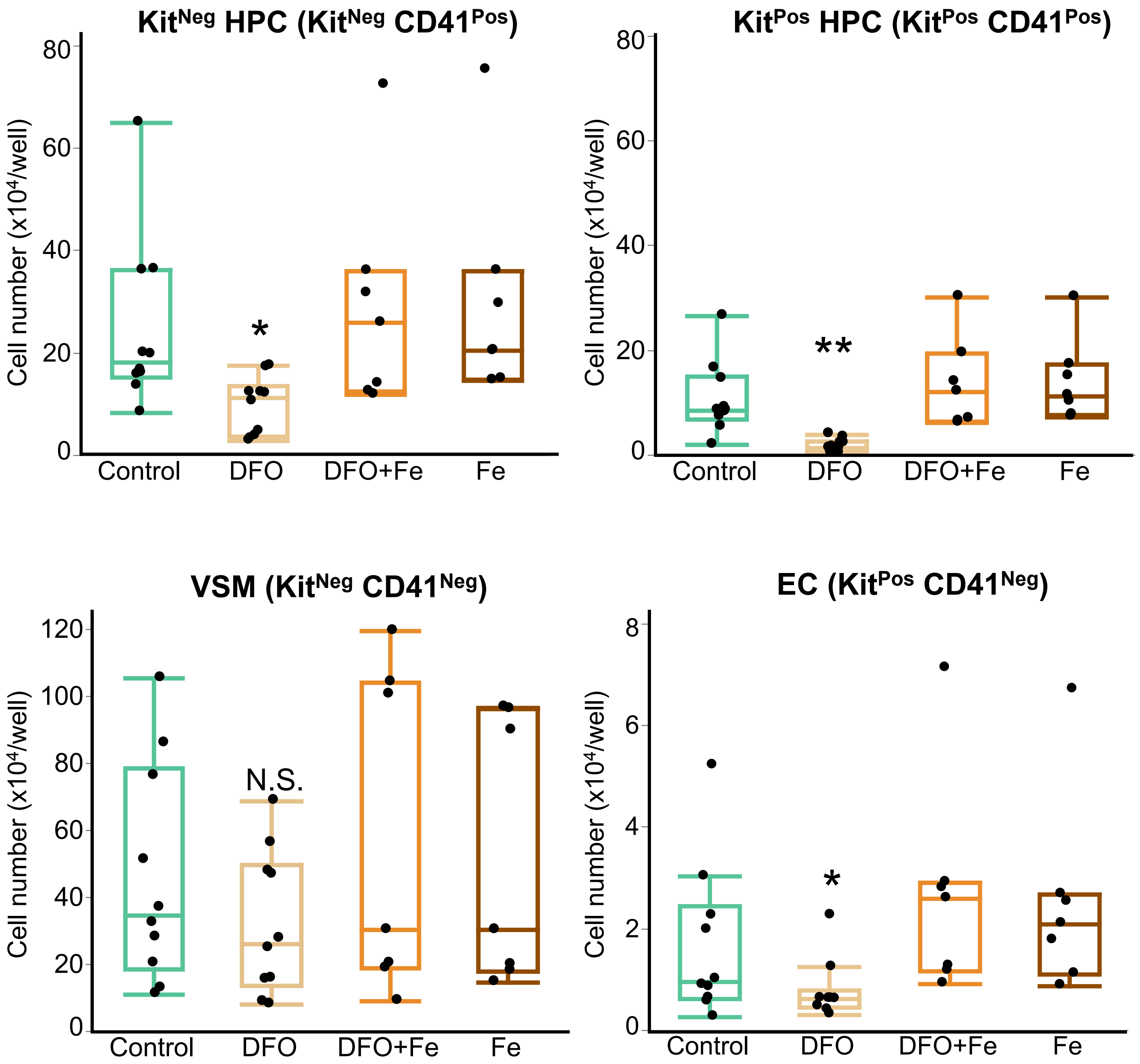

Tukey's boxplots of cell numbers calculated from flow cytometry experiments as cell type frequency * total cell number (10^4 cells/well)/100. Box whiskers show minimum and maximum, the line inside boxes shows median. For control and DFO groups N=10; for DFO+Fe N=7; for Fe N=7. Control versus DFO was analyzed by paired t-test. N.S. = not significant. * Significant at $p<0.05$, ** significant at $p<0.01$.

Supplementary Figure 3: Results of single cell transcriptome analysis

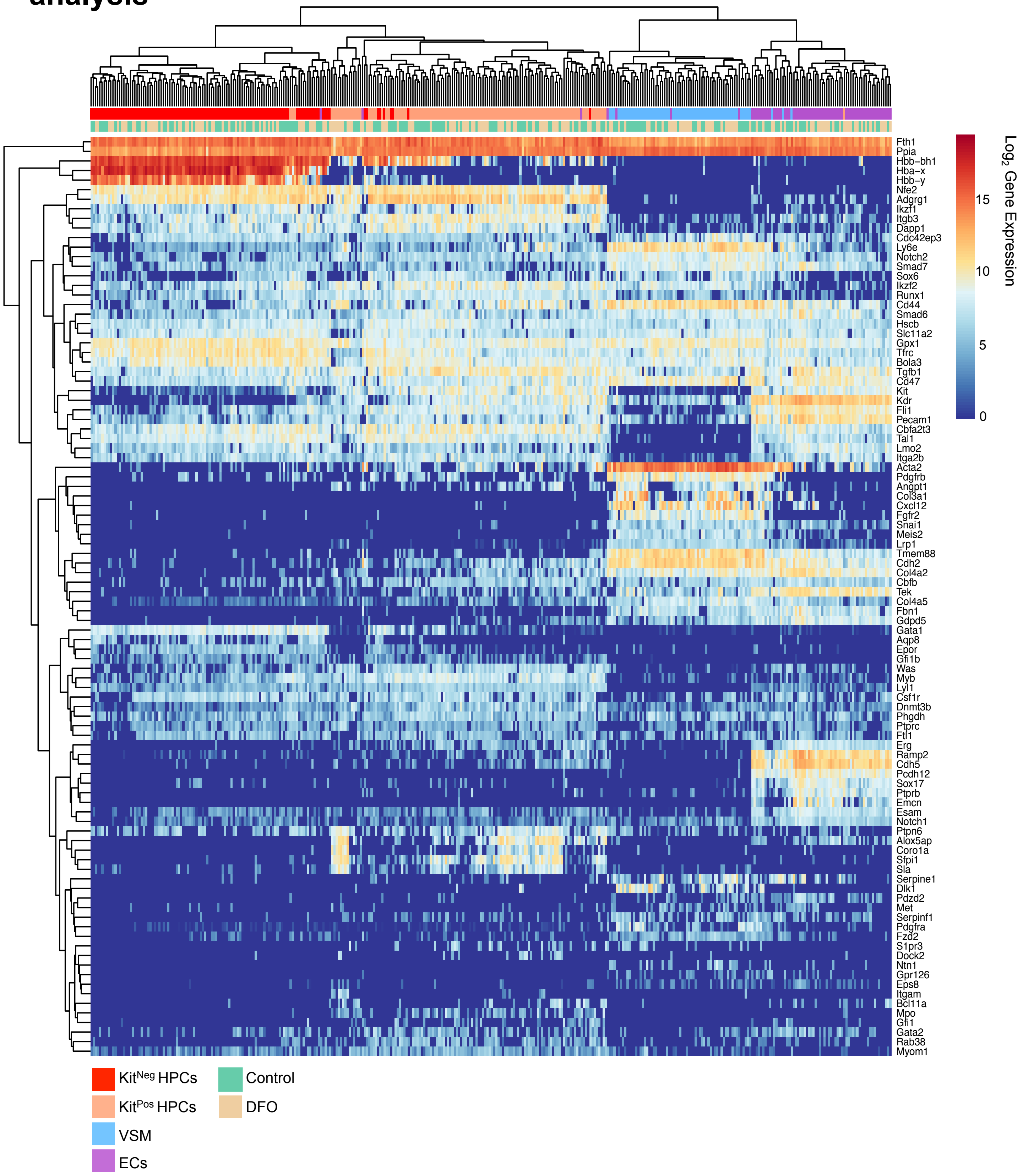

Expression Heatmap showing the 366 cells tested by sc-q-RT-PCR. The four cell clusters and experimental conditions (Control and DFO) are indicated. Two independent experiments were performed.

Supplementary Figure 4: Number of differentially expressed genes between the different cell populations

ANOVA Pairwise Summary

Number of genes with ANOVA P-Value < 0.05 (96 genes in total)

	Control EC	Control Kit^{Neg} HPC	Control Kit^{Pos} HPC	Control VSM	DFO EC	DFO Kit^{Neg} HPC	DFO Kit^{Pos} HPC	DFO VSM
Control EC								
Control Kit^{Neg} HPC	69							
Control Kit^{Pos} HPC	66	50						
Control VSM	48	66	74					
DFO EC	4	66	60	48				
DFO Kit^{Neg} HPC	71	4	51	65	64			
DFO Kit^{Pos} HPC	61	48	13	70	57	53		
DFO VSM	51	65	72	3	48	63	74	

ANOVA pairwise summary showing the number of differentially expressed genes between the indicated eight conditions. Differences between DFO and Control conditions for each of the main populations were relatively small.

Supplementary Table 1: High iron concentration has no significant effect on cell-type frequencies. Frequencies of different cell types in blast culture were measured by flow cytometry. Data are presented as mean ± SEM, N=4, one-way ANOVA and Tukey's multiple comparisons test.

N.S. = not significant Fe 10mM vs Fe 0mM.

**Iron concentration**	**Cell type frequency (%)**			
	VSM	EC	Kit^{Pos} HP	Kit^{Neg} HP
**0 mM**	53.2 ± 9.8	1.1 ± 0.5	11.9 ± 2.5	33.8 ± 7.5
**0.2 mM**	49.8 ± 8.7	1.5 ± 0.3	15.8 ± 3.5	32.9 ± 5.3
**0.5 mM**	49.2 ± 9.7	1.8 ± 0.3	16.7 ± 3.6	32.4 ± 6.1
**1 mM**	48.5 ± 9.1	1.9 ± 0.4	18.1 ± 3.4	31.6 ± 5.7
**5 mM**	53.1 ± 9.5	2.9 ± 0.9	18.8 ± 3.8	25.2 ± 5.5
**10 mM**	63.8 ± 9.2	3.4 ± 0.8	14.7 ± 4.1	18.2 ± 4.5 $^{N.S.}$

Supplementary Table 2: High iron concentration has no significant effect on cell numbers. Data are presented as mean ± SEM, N=4, one-way ANOVA and Tukey's multiple comparisons test.

* Significant Fe 10mM vs Fe 1mM at p=0.026

Significant Fe 10mM vs Fe 1mM at p=0.036

**Iron concentration**	**Cell number (x10^4)**			
	VSM	EC	Kit^{Pos} HP	Kit^{Neg} HP
**0 mM**	29.5 ± 10.4	0.6 ± 0.3	5.2 ± 0.9	14.7 ± 3.1
**0.2 mM**	20.8 ± 5.5	0.6 ± 0.1	5.9 ± 0.8	12.8 ± 1.8
**0.5 mM**	17.7 ± 7.0	0.5 ± 0.2	4.3 ± 0.4	8.7 ± 1.3
**1 mM**	31.2 ± 13.2	0.9 ± 0.3	8.5 ± 1.9	15.5 ± 4.6
**5 mM**	26.3 ± 13.9	1.2 ± 0.5	6.4 ± 1.3	8.1 ± 1.0
**10 mM**	17.6 ± 5.9	0.8 ± 0.2	3.1 ± 0.3 *	3.8 ± 0.2 #

Supplementary Table 3: Iron excess increases cell death only at high Fe concentration. Cell death was measured after 24h of treatment by flow cytometry as a frequency (%) of AnnexinV+ 7AAD+ cells from each cell type. Data are presented as mean ± SE, N=4.

N.S. – not significant Fe vs control

* Significant Fe vs control at p<0.05, one-way ANOVA + Tukey

** Significant Fe vs control at p<0.01, one-way ANOVA + Tukey

Significant Fe 10mM vs Fe 1mM at p<0.05, one-way ANOVA + Tukey

Cell type	Control Fe 0mM (%)	Fe 1mM (%)	Fe 10mM (%)
**VSM**	1.2 ± 0.3	0.9 ± 0.2 (N.S.)	3.5 ± 0.7 *
**EC**	5.3 ± 2.8	3.4 ± 1.2 (N.S.)	7.9 ± 1.0 (N.S.)
**Kit^{Pos} HPCs**	1.4 ± 0.4	1.5 ± 0.5 (N.S.)	3.2 ± 0.5 (N.S.)
**Kit^{Neg} HPCs**	0.6 ± 0.1	0.4 ± 0.1 (N.S.)	1.2 ± 0.3 #

Supplementary Table 4: Iron excess at high concentration leads to increase of preapoptotic cells. Cell death was measured after 24h of treatment by flow cytometry as a frequency (%) of AnnexinV+ 7AAD- cells from each cell type. Data are presented as mean ± SE, N=4.

N.S. – not significant Fe vs control

* Significant Fe vs control at p<0.05, one-way ANOVA + Tukey

** Significant Fe vs control at p<0.01, one-way ANOVA + Tukey

Cell type	Control Fe 0mM (%)	Fe 1mM (%)	Fe 10mM (%)
**VSM**	9.5 ± 1.0	12.1 ± 1.1 (N.S.)	18.5 ± 5.7 (N.S.)
**EC**	9.4 ± 0.7	17.7 ± 1.4 (N.S.)	28.7 ± 4.6 **
**Kit^{Pos} HPCs**	5.7 ± 0.2	11.3 ± 0.6 (N.S.)	14.8 ± 2.8 *
**Kit^{Neg} HPCs**	4.5 ± 0.6	5.2 ± 0.7 (N.S.)	8.2 ± 1.6 (N.S.)

Supplementary Table 5. Iron deficiency reduces proliferation of Kit^{pos} HPCs. Cell proliferation was measured as a frequency (%) of S-phase EdU-positive cells. Data are presented as mean ± SEM, N=4

* Significant DFO vs control at $p<0.05$, one-way ANOVA + Tukey

Cell type	Control	DFO	DFO + Fe
**VSM**	29.5 ± 8.0	9.6 ± 1.9	32.2 ± 10.5
**EC**	23.1 ± 7.8	3.5 ± 1.6	23.2 ± 8.4
**Kit^{Pos} HPCs**	54.4 ± 7.8	18.7 ± 3.2*	50.9 ± 12.5
**Kit^{Neg} HPCs**	64.5 ± 9.1	47.2 ± 4.7	63.9 ± 14.8

Supplementary Table 6: Iron deficiency differentially increases cell death. Cell death was measured by flow cytometry as a frequency (%) of AnnexinV+ 7AAD+ cells from each cell type. Data are presented as mean ± SEM, N=4.

N.S. – not significant DFO vs control

* Significant DFO vs control at $p<0.05$, one-way ANOVA + Tukey

** Significant DFO vs control at $p<0.01$, one-way ANOVA + Tukey

Significant DFO vs Fe treated groups at $p<0.05$, one-way ANOVA + Tukey

DFO treatment time (hours)	Cell type	Control (%)	DFO (%)	DFO + Fe (%)	Fe (%)
**24h**	VSM	4.9 ± 0.8	8.8 ± 2.2 (N.S.)	3.6 ± 0.5	3.8 ± 0.4
**24h**	EC	20.4 ± 3.9	40.7 ± 5.6 *	11.4 ± 2.3	12.4 ± 3.4
**24h**	Kit^{Pos} HPCs	7.2 ± 4.2	17.4 ± 4.9 #	2.3 ± 1	1.5 ± 0.5
**24h**	Kit^{Neg} HPCs	4.1 ± 1.3	9.8 ± 2.2 **	2.5 ± 0.6	1.8 ± 0.3
**48h**	VSM	9.3 ± 1.5	25.5 ± 6.4 *	6.1 ± 0.7	6.6 ± 1.0
**48h**	EC	13.7 ± 6.4	66.2 ± 9.2 **	10.5 ± 2.6	9.2 ± 3.4
**48h**	Kit^{Pos} HPCs	8.1 ± 4.2	73.7 ± 5.8 **	5.1 ± 2.7	5.1 ± 3.0
**48h**	Kit^{Neg} HPCs	8.3 ± 2.3	22.5 ± 3.0 *	3.7 ± 0.8	4.2 ± 0.9

Supplementary Table 7: Iron deficiency does not affect pre-apoptotic cells. The frequency of pre-apoptotic cells was measured by flow cytometry as a frequency (%) of AnnexinV+ 7AAD- cells from each cell type. Data are presented as mean ± SEM, N=4.

N.S. – not significant DFO vs control

Significant DFO vs Fe treated groups at p<0.05, one-way ANOVA + Tukey

DFO treatment time (hours)	Cell type	Control (%)	DFO (%)	DFO + Fe (%)	Fe (%)
**24h**	VSM	14.3 ± 4.4	15.6 ± 4.6 (N.S.)	11.0 ± 2.6	10.0 ± 3.6
**24h**	EC	12.9 ± 4.4	8.8 ± 1.6 (N.S.)	13.1 ± 4.6	13.4 ± 5.7
**24h**	Kit^{Pos} HPCs	27.8 ± 12.6	20.7 ± 8.5 (N.S.)	13.5 ± 8.7	12.0 ± 7.8
**24h**	Kit^{Neg} HPCs	31.9 ± 14.7	28.8 ± 10.6 (N.S.)	17.3 ± 10.8	16.3 ± 11.4
**48h**	VSM	19.4 ± 2.8	21.1 ± 4.2 #	9.3 ± 1.2	9.1 ± 1.2
**48h**	EC	13.3 ± 4.6	2.8 ± 1.6 (N.S.)	9.2 ± 4.0	8.1 ± 1.2
**48h**	Kit^{Pos} HPCs	36.7 ± 18.4	0.8 ± 0.3 (N.S.)	21.5 ± 12.1	19.7 ± 10.6
**48h**	Kit^{Neg} HPCs	25.5 ± 9.7	10.2 ± 3.4 (N.S.)	14.0 ± 6.6	13.5 ± 6.1

Supplementary Table 8: Kit^{Neg} HPCs have higher labile iron compared to other cell types. Cytosolic labile iron levels in control untreated cells were measured with calcein by flow cytometry as described[20]. Data are presented as mean± SEM, N=6 for control, N=4 for treatments, one-way ANOVA and Tukey's multiple comparisons test.

* Significant in Kit^{Neg} HPCs vs other cell types.

$^{\#}$ Significant in Fe vs control, p<0.0001

$^{\$}$ Significant in Fe vs control, p=0.025

Cell type	Relative labile iron content (relative unit)		
	Control	DFO	Fe
**VSM**	0.13 ± 0.03	0.01 ± 0.02	1.00 ± 0.16 $^{\#}$
**EC**	0.20 ± 0.03	0.07 ± 0.03	0.81 ± 0.04 $^{\#}$
**Kit^{Pos} HPCs**	0.19 ± 0.01	0.08 ± 0.04	0.62 ± 0.06 $^{\#}$
**Kit^{Neg} HPCs**	0.37 ± 0.06*	0.23 ± 0.07	0.70 ± 0.10 $^{\$}$
